# Effective management of congenital preauricular sinus to reduce recurrence

**DOI:** 10.6026/973206300223273

**Published:** 2026-06-30

**Authors:** Nitin Kumar Jain, Suryakant Tiwari, Salabh Kumar Agrawal, Abhay Kumar Sinha

**Affiliations:** 1 Department of ENT, Chhindwara Institute of Medical Sciences, Chhindwara, Madhya Pradesh, India

**Keywords:** Preauricular sinus, sinusectomy, supra-auricular approach, recurrence, cosmetic outcome, infection

## Abstract

Congenital preauricular sinus is a common developmental anomaly with frequent recurrence and post-operative complications despite surgical management. Therefore, it is of interest to compare simple sinusectomy and the supra-auricular approach regarding recurrence, infection, operative time, healing, cosmetic outcomes and patient satisfaction. Hence, eighty-six patients were prospectively assigned to either simple sinusectomy (n=42) or supra-auricular excision (n=44) and followed for 12 months. The supra-auricular approach showed zero recurrence, better cosmetic satisfaction and higher patient approval, despite a longer operative time. Thus, we support the supra-auricular approach as the preferred technique for effective and cosmetically superior management of preauricular sinus.

## Background:

Congenital preauricular sinus (PAS) is a common developmental defect resulting from incomplete fusion of the first and second branchial arches during embryogenesis. It typically manifests as a small pit anterior to the ear and may present with recurrent infections, abscess formation, or persistent discharge [1, 2]. The reported incidence varies from 0.1-0.9% in Western populations to up to 4% in Asian and African groups [3]. Surgical excision is the definitive treatment, but recurrence after surgery remains a concern, with rates ranging from 5% to 42% depending on technique [4, 5, 6]. The simple sinusectomy technique, involving excision of the sinus tract under direct visualization, is widely practiced but prone to recurrence due to incomplete removal of branched tracts [7]. In contrast, the supra-auricular approach, offers a broader surgical field and better visualization of deep or branching tracts, potentially lowering recurrence rates [8]. Recent studies have reported significantly improved outcomes with this method [9, 10, 11], while most comparative studies focus only on recurrence, few have systematically analysed other post-operative outcomes such as infection rate, wound healing, pain and cosmetic satisfaction. Comprehensive outcome assessment is essential for determining the overall effectiveness and patient acceptability of surgical techniques. Therefore, it is of interest to compare simple sinusectomy and supra-auricular approach for PAS management, assessing recurrence, infection, operative time, wound healing, pain, cosmetic results, hospital stay and patient satisfaction over a two-year prospective period.

## Methodology:

A prospective comparative study was conducted in the Department of Otorhinolaryngology at Chhindwara Institute of Medical Sciences, Chhindwara (M.P.), from January 2020 to December 2023 after obtaining approval from the Institutional Ethics Committee. A total of 86 patients diagnosed with congenital preauricular sinus were enrolled and randomly allocated into two groups: Group A (n=42) undergoing simple sinusectomy and Group B (n=44) treated with the supra-auricular approach. Patients aged 5 years and above with primary congenital preauricular sinus, whether infected or non-infected, who were willing to participate and comply with a 12-month follow-up were included in the study, while those with recurrent or previously operated cases, associated congenital syndromes, or loss to follow-up before 12 months were excluded. In the simple sinusectomy procedure, an elliptical incision was made around the sinus opening and the tract was delineated using methylene blue followed by complete excision through careful dissection. In the supra-auricular approach, an extended post-auricular incision was made superiorly, skin flaps were elevated to enhance exposure and the sinus tract was dissected under direct visualization up to its termination, including removal of any cartilaginous extensions. Intraoperative parameters such as operative time, measured from skin incision to closure and blood loss, estimated visually and via suction volume, were recorded. Post-operatively, all patients received antibiotics and analgesics for five days and were followed up at 1 week and at 1, 3, 6 and 12 months. Outcome measures included recurrence, defined as reappearance of discharge or sinus opening within 12 months; infection, indicated by wound erythema or discharge within two weeks; pain assessed using the Visual Analogue Scale on day 1 and day 3; wound healing time measured as days to complete epithelialization; cosmetic outcome evaluated using the Patient and Observer Scar Assessment Scale at three months; patient satisfaction assessed on a 10-point Likert scale at six months; and duration of hospital stay. Statistical analysis was performed using SPSS version 25.0, with continuous variables expressed as mean ± standard deviation and categorical variables analyzed using Chi-square test or t-test, considering a p-value of less than 0.05 as statistically significant.

## Results:

A total of 86 patients were enrolled and underwent surgical management of preauricular sinus during the study period. The cohort comprised 48 male and 38 female patients, yielding a male-to-female ratio of 1.26:1. The mean age of the study population was 18.5 ± 6.3 years. Unilateral right-sided involvement was the most common presentation, observed in 61% of cases, while bilateral lesions were noted in 6% of patients. Forty-two patients underwent simple sinusectomy and 44 underwent the supra-auricular approach. All patients completed the 12-month follow-up. Intraoperative parameters differed significantly between the two surgical approaches (Table 1). The mean operative time was significantly longer in the supra-auricular group (47.6 ± 8.5 minutes) than in the simple sinusectomy group (32.4 ± 6.2 minutes; p < 0.001), reflecting the more extensive dissection required to access and excise the deeper tract. Intraoperative blood loss was also significantly higher in the supra-auricular group (12.2 ± 3.6 mL versus 8.0 ± 2.2 mL; p = 0.004), again consistent with the wider surgical exposure. Importantly, however, the proportion of cases requiring concomitant cartilage removal - used here as a surrogate marker for the completeness of tract excision - was significantly greater in the supra-auricular group (38.6% versus 9.5%; p = 0.01), indicating that this approach permitted more thorough removal of the sinus tract along its course toward the auricular cartilage. Postoperative outcomes are summarised in Table 2. Early postoperative pain, assessed by the Visual Analogue Scale, was comparable between groups on Day 1 (4.8 ± 1.2 versus 5.1 ± 1.1; p = 0.24) and on Day 3 (2.9 ± 1.0 versus 2.6 ± 0.9; p = 0.32), with no statistically significant difference at either time point. Infection rates were low and similar between groups (4.8% in the simple sinusectomy group versus 2.3% in the supra-auricular group; p = 0.59). Wound healing time (9.2 ± 1.6 versus 9.8 ± 1.9 days; p = 0.18) and length of hospital stay (1.6 ± 0.5 versus 1.8 ± 0.6 days; p = 0.22) likewise did not differ significantly between the two groups, indicating that the more extensive supra-auricular approach did not translate into delayed recovery or prolonged hospitalisation. Cosmetic and patient-reported outcomes, in contrast, were significantly better in the supra-auricular group. Mean cosmetic satisfaction scores were 8.9 ± 0.7 in the supra-auricular group compared with 7.6 ± 0.9 in the simple sinusectomy group (p < 0.01), and overall patient satisfaction scores were similarly superior (9.1 ± 0.6 versus 7.8 ± 0.8; p < 0.01). On clinical examination, supra-auricular incisions were well concealed within natural skin creases above the helical root, contributing to the more favourable aesthetic outcome. Most importantly, the recurrence rate over 12 months of follow-up was significantly lower in the supra-auricular group, with no recurrences recorded (0%) compared with 2 recurrences (4.8%) in the simple sinusectomy group (p = 0.045). No major complications, including facial nerve injury, keloid formation or hematoma, were observed in either group.

## Discussion:

The present study demonstrates that the supra-auricular approach offers superior outcomes in terms of recurrence prevention and patient satisfaction compared to simple sinusectomy. The recurrence rate of 4.8% in the sinusectomy group and 0% in the supra-auricular group closely matches rates reported in earlier studies- El-Anwar and El-Aassar [8] reported 0% recurrence using the supra-auricular approach, while Leopardi *et al.* (2017) [9] found recurrence rates between 2% and 5%. This reduction is attributable to better exposure of the tract, permitting complete excision of branches extending towards cartilage or temporalis fascia [10]. Although the supra-auricular approach required a longer operative time (approx. 15 minutes more), this trade-off is acceptable considering the significant reduction in recurrence. Both groups showed good wound healing, highlighting that meticulous technique and post-operative care are more critical than incision type for preventing infection. Pain scores were comparable between groups, with most patients reporting only mild discomfort by day 3. Cosmetic and satisfaction scores were significantly higher in the supra-auricular group due to hidden incisions and reduced recurrence, in agreement with recent studies emphasizing patient-centered outcomes [10, 11 and 12]. No major complications occurred, confirming that the supra-auricular approach, when performed carefully, is safe and preserves facial nerve integrity.

## Conclusion:

The supra-auricular approach is superior to simple sinusectomy for the management of congenital preauricular sinus. It significantly reduces recurrence while providing better cosmetic outcomes and higher patient satisfaction. Despite more operative time and blood loss, it remains the most effective and reliable technique for complete sinus excision with minimal morbidity.

## Figures and Tables

**Table 1:** Comparison of intraoperative outcomes

**Parameter**	**Simple sinusectomy (n=42)**	**Supra-Auricular (n=44)**	**p-value**
Operative time (min)	32.4 ± 6.2	47.6 ± 8.5	<0.001*
Blood loss (mL)	8.0 ± 2.2	12.2 ± 3.6	0.004*
Cartilage removal (%)	9.50%	38.60%	0.01*
*Significant

**Table 2:** Comparison of post-operative outcomes

**Outcome**	**Simple sinusectomy**	**Supra-Auricular**	**p-value**
Pain (VAS Day 1)	4.8 ± 1.2	5.1 ± 1.1	0.24
Pain (VAS Day 3)	2.9 ± 1.0	2.6 ± 0.9	0.32
Infection rate	2 (4.8%)	1 (2.3%)	0.59
Wound healing (days)	9.2 ± 1.6	9.8 ± 1.9	0.18
Hospital stay (days)	1.6 ± 0.5	1.8 ± 0.6	0.22
Cosmetic satisfaction (0-10)	7.6 ± 0.9	8.9 ± 0.7	<0.01*
Recurrence rate	2 (4.8%)	0 (0%)	0.045*
Patient satisfaction (0-10)	7.8 ± 0.8	9.1 ± 0.6	<0.01*
*Statistically significant difference
